# CUB Domain-Containing Protein 1 (CDCP1) is a rational target for the development of imaging tracers and antibody-drug conjugates for cancer detection and therapy

**DOI:** 10.7150/thno.78171

**Published:** 2022-10-03

**Authors:** Tashbib Khan, Nicholas J. Lyons, Madeline Gough, Kayden K.X. Kwah, Tahleesa J. Cuda, Cameron E. Snell, Brian W. Tse, Kamil A. Sokolowski, Lesley A. Pearce, Timothy E. Adams, Stephen E. Rose, Simon Puttick, Marina Pajic, Mark N. Adams, Yaowu He, John D. Hooper, Thomas Kryza

**Affiliations:** 1 Mater Research Institute - The University of Queensland, Translational Research Institute, 37 Kent Street, Woolloongabba, QLD, Australia.; 2 Mater Health Services, South Brisbane, QLD, Australia.; 3 Preclinical Imaging Facility, Translational Research Institute, Woolloongabba, QLD, Australia.; 4 Commonwealth Scientific and Industrial Research Organisation Manufacturing, Parkville, VIC, Australia.; 5 Commonwealth Scientific and Industrial Research Organisation, Herston, QLD, Australia.; 6 The Kinghorn Cancer Centre, Garvan Institute of Medical Research, Faculty of Medicine, St Vincent's Clinical School, University of New South Wales, Sydney, NSW, Australia.; 7 School of Biomedical Sciences, Centre for Genomics and Personalised Health, Queensland University of Technology, Brisbane, QLD, Australia.

**Keywords:** CDCP1, cancer, theranostics, antibody-drug conjugate, receptor

## Abstract

**Rationale:** An antibody-drug conjugate (ADC) is a targeted therapy consisting of a cytotoxic payload that is linked to an antibody which targets a protein enriched on malignant cells. Multiple ADCs are currently used clinically as anti-cancer agents significantly improving patient survival. Herein, we evaluated the rationale of targeting the cell surface oncoreceptor CUB domain-containing protein 1 (CDCP1) using ADCs and assessed the efficacy of CDCP1-directed ADCs against a range of malignant tumors.

**Methods:** CDCP1 mRNA expression was evaluated using large transcriptomic datasets of normal/tumor samples for 23 types of cancer and 15 other normal organs, and CDCP1 protein expression was examined in 34 normal tissues, >300 samples from six types of cancer, and in 49 cancer cell lines. A recombinant human/mouse chimeric anti-CDCP1 antibody (ch10D7) was labelled with ^89^Zirconium or monomethyl auristatin E (MMAE) and tested in multiple pre-clinical cancer models including 36 cancer cell lines and three mouse xenograft models.

**Results:** Analysis of CDCP1 expression indicates elevated CDCP1 expression in the majority of the cancers and restricted expression in normal human tissues. Antibody ch10D7 demonstrates a high affinity and specificity for CDCP1 inducing cell signalling via Src accompanied by rapid internalization of ch10D7/CDCP1 complexes in cancer cells*.* ^89^Zirconium-labelled ch10D7 accumulates in CDCP1 expressing cells enabling detection of pancreatic cancer xenografts in mice by PET imaging. Cytotoxicity of MMAE-labelled ch10D7 against kidney, colorectal, lung, ovarian, pancreatic and prostate cancer cells *in vitro*, correlates with the level of CDCP1 on the plasma membrane. ch10D7-MMAE displays robust anti-tumor effects against mouse xenograft models of pancreatic, colorectal and ovarian cancer.

**Conclusion:** CDCP1 directed imaging agents will be useful for selecting cancer patients for personalized treatment with cytotoxin-loaded CDCP1 targeting agents including antibody-drug conjugates.

## Introduction

Personalized therapies that target the distinct oncogenic features of patient tumors are promising innovations in the war against cancer 1-4. A growing number of theranostic agents and antibody-drug conjugates (ADCs) are now in clinical use for personalised management of cancer 5, 6. The proteins targeted by these agents conform to a set of criteria including elevated expression on the surface of malignant cells, restricted normal tissue expression, and the availability of high affinity, high specificity targeting agents including antibodies 1, 5, 6. It is also generally necessary for ADCs to be internalized by target cells to optimize the activity of cytotoxic payloads 1.

A recent example of successful personalization is the targeting of prostate-specific membrane antigen (PSMA) in prostate cancer. In the clinical setting, receptor-targeted positron-emission tomography (PET) imaging first defines avidity of ^68^Galium-PSMA ligands for tumors. For patients who have sufficiently PET-avid tumors, imaging is followed by treatment using the same PSMA ligand conjugated to the therapeutic radionuclide ^177^Lutetium (^177^Lu) 7, 8. This approach has particularly benefited prostate cancer patients who have castrate-resistant metastatic disease where responses to conventional therapies are poor 9. The success of PSMA in prostate cancer, and the ongoing broader clinical need for therapeutic options for otherwise treatment-resistant malignancies, has driven interest in developing novel personalized receptor targeting agents for a range of cancers 3.

CUB-domain containing protein 1 (CDCP1) is a cell surface glycoprotein that is elevated in a range of malignancies and mediates oncogenic processes promoting cancer cell survival, growth, metastasis and treatment resistance 10. CDCP1 regulates these processes via intersection with pro-oncogenic molecular pathways mediated by signalling mediators such as Src, MAPK, PI3K/AKT, EGFR and integrins 10. Activation of these pathways occurs through full-length CDCP1 (CDCP1-FL) and via CDCP1 that has undergone proteolysis at the cell surface generating amino- (CDCP1-ATF) and carboxyl-terminal (CDCP1-CTF) fragments 10. These signalling events via CDCP1 are generally accompanied by its phosphorylation at residue tyrosine 734 (Y734) 10. CDCP1-ATF is detectable in the serum of colorectal cancer patients 11 but appears to remain predominantly on the plasma membrane tethered to CDCP1-FL or CDCP1-CTF 12, 13.

Preclinical data support CDCP1 as a target for delivery of imaging and cytotoxic payloads for detection and personalized treatment of cancers that display elevated levels of this receptor and currently have limited treatment options for advanced disease including ovarian clear cell carcinoma, high-grade serous ovarian carcinoma, pancreatic ductal adenocarcinoma (**PDAC**), colorectal cancer and castration resistant prostate cancer including PSMA null disease 12-16. Mouse monoclonal antibody 10D7, directed against an epitope within amino acids 30 to 358 of CDCP1-ATF, binds with high affinity to induce rapid internalization of receptor/antibody complexes, is effective at delivering ^89^Zirconium (**^89^Zr**) and monomethyl auristatin E (**MMAE**) for, respectively, PET-based detection and treatment of cell line and patient-derived xenograft ovarian cancer models in mice 15. This antibody is also highly effective at delivering radionuclide ^89^Zr for PET detection of cell line and patient-derived xenografts in mice of colorectal cancer 14 and PDAC 12, and cytotoxin MMAE for treatment *in vivo* of PDAC models in mice achieving anti-tumor effects superior to chemotherapy 12. Successful *in vivo* targeting of preclinical models of PDAC has also been achieved with ^89^Zr- and MMAE-linked recombinant human antibody 4A06 against the CDCP1 ectodomain 17. Antibody A406 was also effective at delivering ^89^Zr for detection and ^177^Lu for radio-ligand therapy of CDCP1 expressing prostate cancer xenografts in mice 16. Most recently, antibody IgG-CL03 directed to the CDCP1-ATF, proximal to protease cleavage sites at ^368^Arg and ^369^Lys, and labelled with ^89^Zr showed strong accumulation in subcutaneous xenografts of PDAC PL5 cells in mice 13. These data demonstrate that CDCP1 targeted antibodies are effective at delivering payloads for *in vivo* detection and treatment of preclinical models of cancer.

In preparation for clinical studies, we report here the bioengineering and evaluation of a human/mouse chimeric form of antibody 10D7, designated ch10D7. In comparison with the parent 10D7 antibody, we determine the affinity of ch10D7 and assess its specificity and perform cell-based assays and experiments in mice to examine its ability to deliver ^89^Zr and MMAE for preclinical detection and treatment, respectively, of several cancers. We also examine CDCP1 expression in a broad range of cancers and 34 normal human tissues to provide insight into the proportion of patients who could benefit from CDCP1 targeted theranostics and ADCs. The described approaches verify the potential of targeting CDCP1 in a range of solid cancers and represent a pipeline for assessing the suitability of receptors as targets for cancer directed theranostics and ADCs.

## Results

### Human/mouse ch10D7 retains the binding affinity of murine 10D7

**Figure 1:**
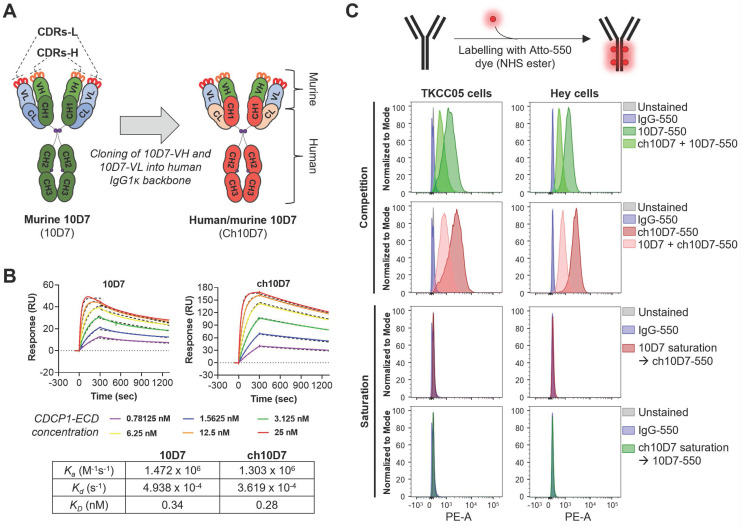
** Generation of human/mouse chimeric anti-CDCP1 antibody ch10D7 from mouse monoclonal antibody 10D7. A.** Schematic illustrating the generation of ch10D7 from murine 10D7. **B.** Comparative analysis of ch10D7 and 10D7 binding affinity to CDCP1-ECD by SPR. Top: SPR-derived sensograms of ch10D7 and 10D7 binding to various concentrations of recombinant CDCP1-ECD. Bottom: Table summarizing association (*K_a_*), dissociation (*K_d_*) and affinity (*K_D_*) constants of ch10D7 and 10D7 to CDCP1-ECD. **C.** Comparison of binding of ch10D7 and 10D7, fluorescently labelled with the dye Atto-550, to PDAC (TKCC05) and ovarian cancer (HEY) cells by flow cytometry. Top panel: Competition experiments in which cells were incubated with either one labelled antibody or the combination of one labelled antibody with the other unlabelled antibody (ratio 1:1). Bottom panel Saturation experiments in which CDCP1 binding sites for one labelled antibody were blocked with saturating amounts of the other unlabelled antibody (ratio 1:10). CDR, Complementarity-determining region; -L, Light; -H, Heavy; VL, Variable light; VH, Variable heavy; CH, Constant heavy; CL, Constant light.

To develop anti-CDCP1 antibody 10D7 for use in clinical studies, we engineered its murine variable heavy (VH) and light (VL) chains onto a human IgG1κ backbone, generating human/mouse chimeric antibody ch10D7 (Figure 1A and S1). To assess the impact of engineering on antibody affinity, we determined the binding kinetics of ch10D7, in comparison with 10D7, to the recombinant extracellular domain of CDCP1 (CDCP1-ECD) by surface plasmon resonance (SPR) spectroscopy. The binding of ch10D7 to CDCP1-ECD displays affinity (K_D_) of 0.28 nM, comparable to that of murine 10D7 of 0.34 nM (Figure 1B). Binding of ch10D7 compared to 10D7 was also examined by flow cytometry analysis of TKCC05 PDAC cells and HEY high-grade serous ovarian cancer cells using the antibodies labelled with the fluorophore Atto-550 (Figure S2). As shown in Figure 1C (top), competition with an equal amount of unlabelled ch10D7 halved the accumulation of fluorophore-labelled 10D7-550 on the surface of cancer cells. Similar results were seen in the reverse assay format where an equal amount of unlabelled 10D7 halved the accumulation of fluorophore-labelled ch10D7-550 on the surface of cancer cells (Figure 1C, second top) supporting that ch10D7 binds with similar affinity to CDCP1 as 10D7. Consistent with this, as assessed by flow cytometry, ch10D7-550 and 10D7-550 were unable to bind to CDCP1 expressing cells in assays in which antibody binding sites on CDCP1 expressing cells had been saturated with 10-fold excess of unlabelled competing antibody 10D7 and ch10D7, respectively (Figure 1C, second bottom and bottom). These data demonstrate that ch10D7 and 10D7 display similar binding to recombinant CDCP1-ECD and CDCP1 expressing cancer cell lines.

### ch10D7 initiates internalisation of CDCP1 in cancer cells *in vitro*

**Figure 2:**
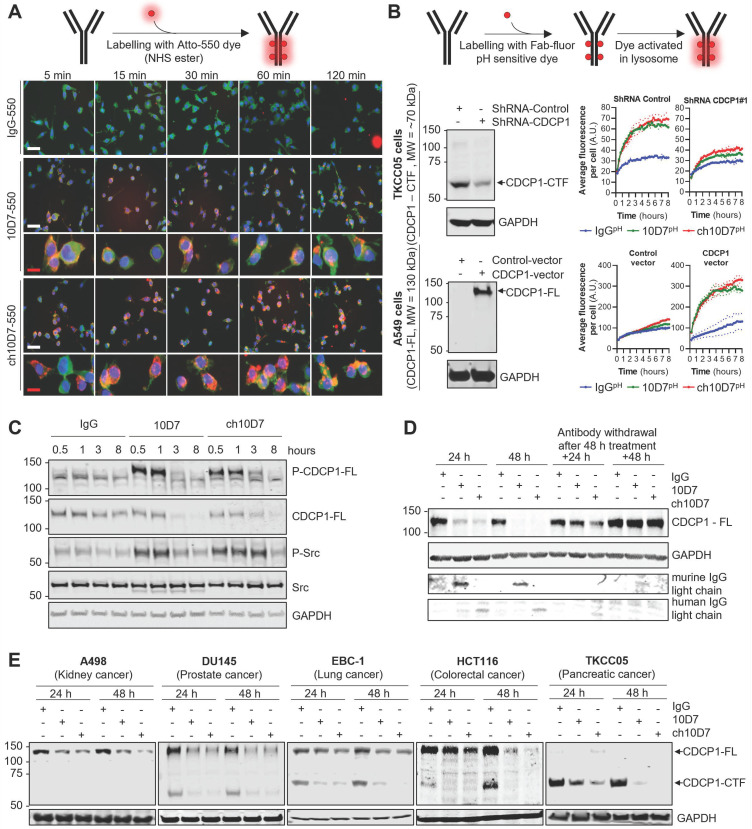
** Antibody ch10D7 induces transient activation of CDCP1 signalling and receptor internalisation and degradation. A.** Top: Schematic illustrating the labelling of antibodies with Atto-550 fluorescent dye. Bottom: Time lapse images (5 -120min) of fluorescently labelled antibodies (5 µg/ml; IgG-, 10D7- or ch10D7-550, red) binding to PDAC TKCC05 cells. After the indicated periods, cells were fixed and counter stained with WGA-488 (membrane staining, green) and DAPI (DNA, blue). White scale bar = 200 µm; Red scale bar = 50 µm. **B.** Assessment of anti-CDCP1 antibody internalization in cancer cells lines that have different levels of CDCP1. Top panel: 10D7, ch10D7 or control IgG were labelled with a Fab fluor conjugated to a pH-sensitive fluorescent dye as depicted. Bottom panel: Internalization of antibodies was assessed in CDCP1 positive PDAC TKCC05 cells stably transduced with a control shRNA or a CDCP1 silencing shRNA (CDCP1-shRNA) and in CDCP1 negative lung cancer A549 cells stably transduced with a control vector or a CDCP1-encoding vector. Internalization was assessed by measurement of the accumulation of fluorescent signal per cell using an Incucyte S3 system after treatment of cells with the labelled antibody (5 µg/ml). For both cellular models, western blot analysis of CDCP1 level are shown on the left. **C and D.** Impact of anti-CDCP1 antibodies on CDCP1 in ovarian cancer cells. **C.** Western blot analysis, using antibodies against p-CDCP1-Y734, CDCP1, p-Src-Y416, Src and GAPDH, of lysates from HEY cells treated for up to 8 h with ch10D7, 10D7 or control IgG (5 µg/ml) . **D.** Western blot analysis, using antibodies against p-CDCP1-Y734, CDCP1, p-Src-Y416, Src and GAPDH, of lysates from HEY cells treated for 24h or 48 h with antibody 10D7, ch10D7 or control IgG (5 µg/ml) before antibody washout then further growth up to 48h in normal medium. **E.** Impact of anti-CDCP1 antibody on CDCP1 expression in five cancer cell lines. Western blot analysis of lysates from cell lines treated for 24 or 48 h with 10D7, ch10D7 or control IgG (5 µg/ml). Lysates were probed by western blot analysis for CDCP1 (antibody 4115) and GAPDH.

ADC therapy presents a potent means of eliciting selective cytotoxicity by binding to plasma membrane targets enriched on cancer cells to deliver a cytotoxic payload. The key requirement for this approach is a biomolecule, such as an antibody, that on binding to its target receptor, induces internalisation and degradation of the receptor-biomolecule complex, so that payloads attached to the antibody are released by the actions of proteases including lysosomal peptidases 1, 18, 19. We have previously shown that mouse 10D7 induces internalisation and subsequent degradation by lysosomal and proteasomal proteases of CDCP1/10D7 complexes in high grade serous ovarian cancer cell lines 15. Thus, we next examined whether ch10D7 exhibits the same properties in cell lines from a range of cancers. To qualitatively assess antibody internalisation, we performed fluorescence microscopy analysis of cells treated for defined time periods with ch10D7, 10D7 or control IgG conjugated to fluorophoreAtto-550. Figure 2A shows that ch10D7-550, as for 10D7-550, is rapidly internalized by TKCC05 PDAC cells with membrane staining apparent at 5 min after initiation of treatments and intracellular staining detected from 15 min and accumulating up to 120 min. In contrast, no signal was apparent from cells incubated with IgG-550 (Figure 2A). To quantify internalisation, TKCC05 PDAC cells stably silenced for CDCP1 and shRNA control TKCC05 cells (Figure 2B, left top), and lung adenocarcinoma A549 cells stably over-expressing CDCP1 and vector control A549 cells (Figure 2B, left bottom), were treated with antibodies labelled with a Fab-fluor pH-sensitive dye then imaged by time-lapse microscopy to monitor accumulation of cellular fluorescence as a measure of antibody internalization. As shown in Figure 2B (top right), CDCP1 expressing TKCC05 cells treated with ch10D7^pH^ or 10D7^pH^ display similar rapid increases in fluorescence, with signal reduced to almost background levels in CDCP1 silenced cells, demonstrating the specificity of this antibody for its target. Also supporting the specificity of ch10D7 for CDCP1, A549 cells which lack endogenous CDCP1, displayed background levels of fluorescence in response to ch10D7^pH^ or 10D7^pH^, with signal markedly and rapidly increasing in cells overexpressing CDCP1 (Figure 2B, bottom right). The results indicate that ch10D7 can induce internalization CDCP1/antibody complexes as effectively as 10D7.

To assess the capacity of ch10D7 to induce CDCP1 downstream signalling, as previously observed for 10D7 12, western blotting was performed for phosphorylated (p)-CDCP1-Y734 and a downstream transducer of CDCP1-mediated signalling p-Src-Y416 10. These phospho-proteins were analyzed in lysates from HEY high grade serous ovarian cancer cells treated with ch10D7,10D7 or IgG-k1 control (5 µg/ml) for 30 minutes to 8 h. Figure 2C shows that ch10D7 and 10D7, but not control IgG, induce marked transient increased levels of p-CDCP1-Y734 and p-Src-Y416. Increased signal was apparent within 0.5 h of initiation of ch10D7 and 10D7 treatments which was sustained for 1 h with signal reducing by 3 h with loss of signal by 8 h (Figure 2C). The reduction in p-CDCP1-Y734 and p-Src-Y416 at 3 h and then loss from 8 h, corresponded with reduction then loss of total CDCP1 caused by ch10D7 and 10D7 treatments (Figure 2C) which was consistent with antibody-induced receptor degradation reported previously 12, 15.

We next assessed the impact of extended periods of antibody exposure then withdrawal, as an indicator of how CDCP1 levels may respond to circulating agents used clinically. CDCP1 levels were examined in lysates from HEY cells treated with the antibodies for 24 and 48 h, and examined after antibody withdrawal for further periods of 24 and 48 h. The analysis was performed with antibody 4115 against the intracellular carboxyl-terminal of CDCP1 which detects both 135 kDa CDCP1-FL and 70 kDa CDCP1-CTF 15. As shown in Figure 2D, the levels of CDCP1-FL expressed by HEY cells were significantly reduced after 24 h and completely lost after 48 h treatment with ch10D7- and 10D7. Re-expression of CDCP1 was apparent 24 h after withdrawal of ch10D7 and 10D7, with receptor levels returned to control levels 48 h after antibody withdrawal (Figure 2D, right). To assess whether antibody-induced loss of CDCP1 expression is a general phenomenon, we also examined the effect of these antibody treatments by western blotting lysates from 14 other cell lines from six different cancers (kidney, prostate, lung, colorectal, pancreatic, ovarian) that express only full-length CDCP1-FL (A498, 786-O, A549, HT29), a mixture of CDCP1-FL and CDCP1-CTF (DU145, EBC-1, HCT116, CAOV3, H1650, H1975, OVCAR420, TKCC23, TKCC2.1), or only CDCP1-CTF (TKCC05). As shown in Figure 2E and S3, in all cell lines ch10D7 caused gradual reduction in levels of CDCP1-FL and CDCP1-CTF that was sustained out to 48 h. The results suggest that ch10D7 will be effective for delivering payloads for internalization by CDCP1 expressing cells.

### Cytotoxin loaded ch10D7 and 10D7 are equally effective against cell lines *in vitro*

**Figure 3:**
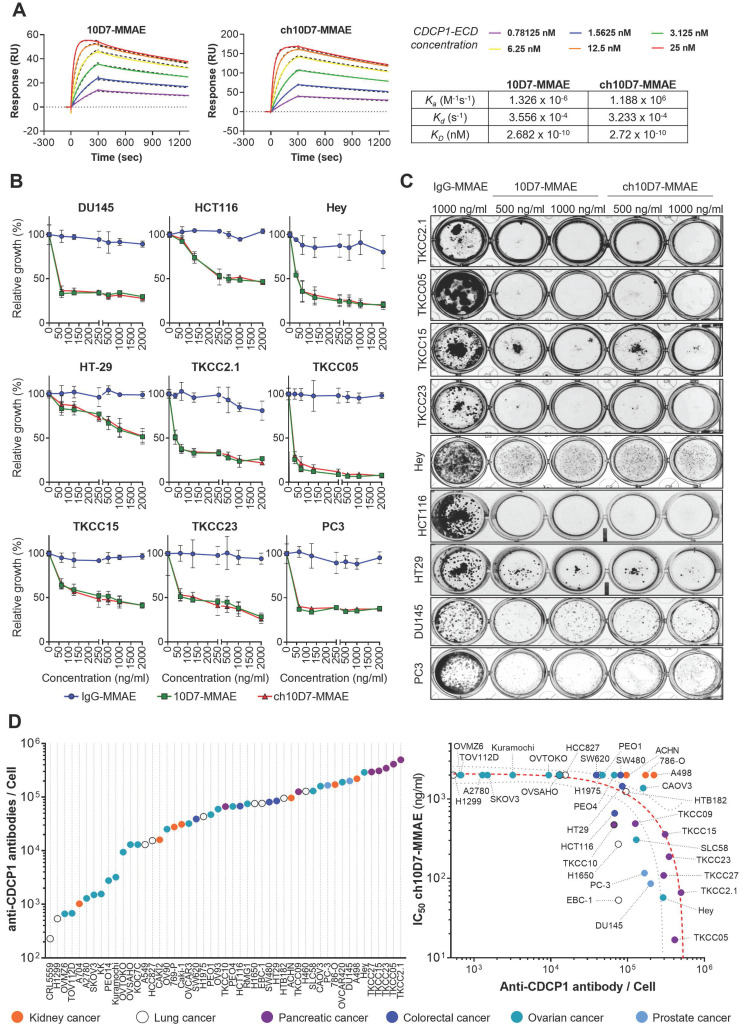
***In vitro* cytotoxicity of anti-CDCP1 ADCs: A.** Comparative analysis of ch10D7-MMAE and 10D7-MMAE ADC binding affinity to CDCP1 ECD by SPR analysis. Top: SPR-derived sensograms of ch10D7-MMAE and 10D7-MMAE binding to various concentrations of recombinant CDCP1-ECD. Bottom: Table summarizing association (K_a_), dissociation (K_d_) and affinity (K_D_) constants of ch10D7-MMAE and 10D7-MMAE to CDCP1-ECD. **B.** Quantitative analysis of growth inhibition of cancer cells by ADCs. Cancer cells (4,000 cells/well) were treated for 6 h with the respective ADC (0 - 2000 ng/ml) then grown for a further 72 h in complete medium. Cell growth was quantified by absorbance measurements at 490 nm of wells incubated with the CellTiter AQueous One Solution Reagent. Data are presented as mean of relative cell growth (compared to untreated cells) +/- SD from three independent experiments. **C.** Qualitative analysis of the impact of ADCs on cancer cell colony formation. Cell lines were treated for 6 h with the respective ADC (500 - 1000 ng/ml) before plating at low density (500 cells/well) in complete medium and grown for a further 10 to 14 days when colonies were fixed using PFA and stained with crystal violet. Representative images of colonies are shown. **D.** Examination of correlation between cell surface CDCP1 and cell response to ADC ch10D7-MMAE. Left: The number of fluorescently labelled anti-CDCP1 antibodies bound per cell was evaluated by flow cytometry against a panel of 49 cancer cell lines using an anti-CDCP1 antibody conjugated with PE (CD318-PE). The number of fluorescently labelled anti-CDCP1 antibodies bound/cell was interpolated from a standard curve generated from known numbers of Quantibright beads. Results are expressed as median of antibodies/cell from at least 5,000 cells. Right: Correlation between the potency of ch10D7-MMAE ADC (represented by GI50 values for each cell line) versus the number of fluorescently labelled anti-CDCP1 antibodies bound/cell. MW, Molecular weight.

We next sought to assess the effect of cytotoxic payloaded ch10D7 on growth of a range of cancer cell lines *in vitro*. Antibodies and IgG control were conjugated to the microtubule disrupting toxin MMAE using a proteolytically-cleavable linker as previously described 12, 15. To ensure that the payload did not impact target binding, the affinity of ch10D7-MMAE for CDCP1-ECD, in comparison with 10D7-MMAE, was determined by SPR spectroscopy. As shown in Figure 3A, ch10D7-MMAE and 10D7-MMAE had the same affinity for CDCP1-ECD and comparison with results in Figure 1B indicated that antibody affinity was unaffected by the MMAE payload.

We evaluated the cytotoxicity of ch10D7-MMAE in comparison with 10D7-MMAE and IgG-MMAE against nine cell lines *in vitro*, with cell viability quantified by measuring cellular metabolism, and assessed qualitatively by examining cell colonies that were able to form after the treatments. As shown in Figure 3B, the viability of the nine cell lines was reduced by at least 50% by ch10D7-MMAE which has the same potency as 10D7-MMAE. These effects on cell viability were consistent with the ability of ch10D7-MMAE and 10D7-MMAE to markedly inhibit colony formation of each of the nine cell lines (Figure 3C).

We next assessed the level of cell surface CDCP1 in 49 cell lines from a diverse range of adenocarcinomas, including seven from kidney, nine from lung, four from colorectal, 20 from ovarian and two from prostate cancer and seven from PDAC. As shown in Figure 3D (left), flow cytometry analysis indicated that cell surface CDCP1 levels vary widely between cancer cell lines, ranging from ~2x10^3^ anti-CDCP1 antibodies bound/cell for lung cancer CRL5559 cells to ~3x10^5^ anti-CDCP1 antibodies bound/cell for PDAC TKCC2.1 cells. Cell surface CDCP1 levels were largely independent of the cancer of origin although highest levels were apparent in five of the seven PDAC lines (Figure 3D; purple). Finally, we investigated if there was any correlation between the number of anti-CDCP1 antibodies bound/cell (from Figure 3D left) and the IC_50_ of ch10D7-MMAE for each cell-line (from Figure 3B and S4). This analysis revealed a trend that higher ch10D7-MMAE efficacy correlates with higher cell surface levels of CDCP1 (Figure 3D right). Of note, cell lines with fewer than 5x10^4^ anti-CDCP1 antibodies bound/cell were largely unresponsive to ch10D7-MMAE suggesting that this would be the lower limit to predict anti-CDCP1 ADC efficacy. However, it is important to note that several cell lines were unresponsive to this CDCP1 targeted ADC despite having anti-CDCP1 antibodies bound/cell levels well above the threshold including kidney cancer A498, 786-O, and ACHN cells, potentially indicating that this type of cancer has a mechanism to avoid ch10D7-MMAE cytotoxicity (Figure 3D right). These results suggest that the level of cell surface receptor expression is important, but not the only variable impacting the efficacy of the CDCP1 targeted ADC ch10D7-MMAE.

### ch10D7 accumulates in xenograft tumors *in vivo*

**Figure 4:**
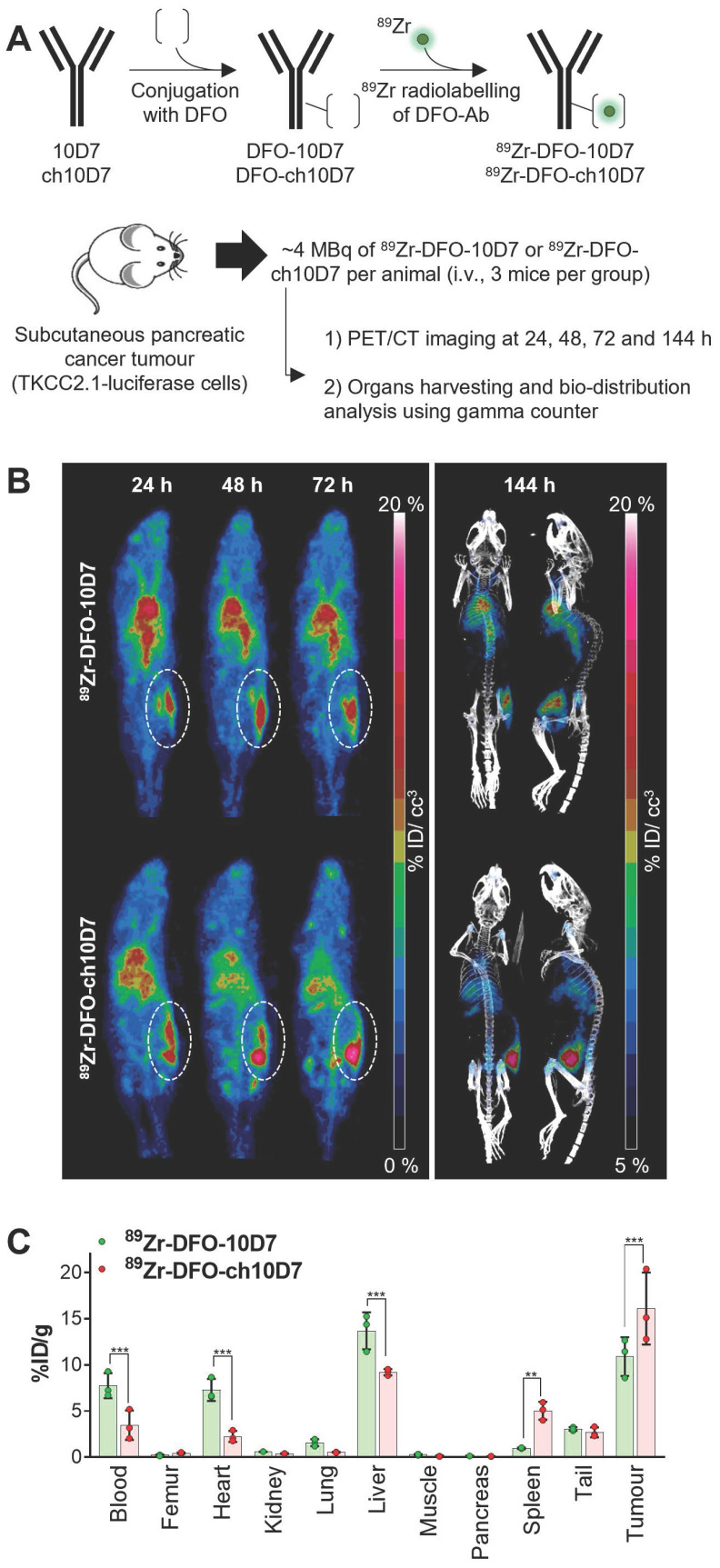
** Anti-CDCP1 antibody ch10D7 accumulates in CDCP1 expressing cancer tissues *in vivo*. A.** Schematic depicting generation of radiolabelled ch10D7-^89^Zr and 10D7-^89^Zr (top), and the experimental plan for the *in vivo* PDAC model (bottom). **B.** Representative PET-CT images of NSG mice carrying subcutaneous xenografts of PDAC TKCC2.1 cells. Left: PET imaging (ventral view maximum intensity projection) at 24, 48 and 72 h after antibody injection. Right: PET/CT imaging (ventral and lateral views maximum intensity projection) at 144 h after antibody injection. **C.** Quantitative distribution analysis of ^89^Zr-DFO-10D7 and ch10D7-^89^Zr 144 h post injection (n = 3) in normal tissues and xenografts. Statistical significance between different groups was performed using a two-way ANOVA test with *** p<0.001.

To assess the capacity of ch10D7 to accumulate in tumors, we performed PET/CT imaging of mice carrying subcutaneous pancreatic tumors after administration of radiolabelled antibodies. ch10D7 and 10D7 conjugated with deferoxamine (DFO) and radiolabelled with ^89^Zr using our previously published protocols (Figure 4A) were injected intravenously into mice (~4MBq/mouse) xenografted with PDAC TKCC2.1 cells which express high cell surface levels of CDCP1 (Figure 3D). PET/CT imaging performed 24, 48, 72 and 144 h later as previously described 12, 14, 15 demonstrated that both ch10D7-^89^Zr and 10D7-^89^Zr accumulate in tumors within 24 h with signal increasing up to 144 h (Figure 4B). As shown in Figure 4C, quantitative biodistribution analysis of recovered organs indicated that ch10D7-^89^Zr accumulated significantly more in tumors than 10D7-^89^Zr at 16.1 versus 10.9 %ID/g while off-tumor levels of 10D7-^89^Zr higher than ch10D7-^89^Zr in the blood (7.7 versus 3.5 %ID/g), heart (7.3 versus 2.2 %ID/g) and liver (13.7 versus 9.2 %ID/g) but lower in spleen (0.9 versus 5.0 %ID/g). The results demonstrate that ch10D7 and 10D7 are able to direct payloads to tumor tissues *in vivo*.

### ch10D7-MMAE is effective against *in vivo* cancer models

**Figure 5:**
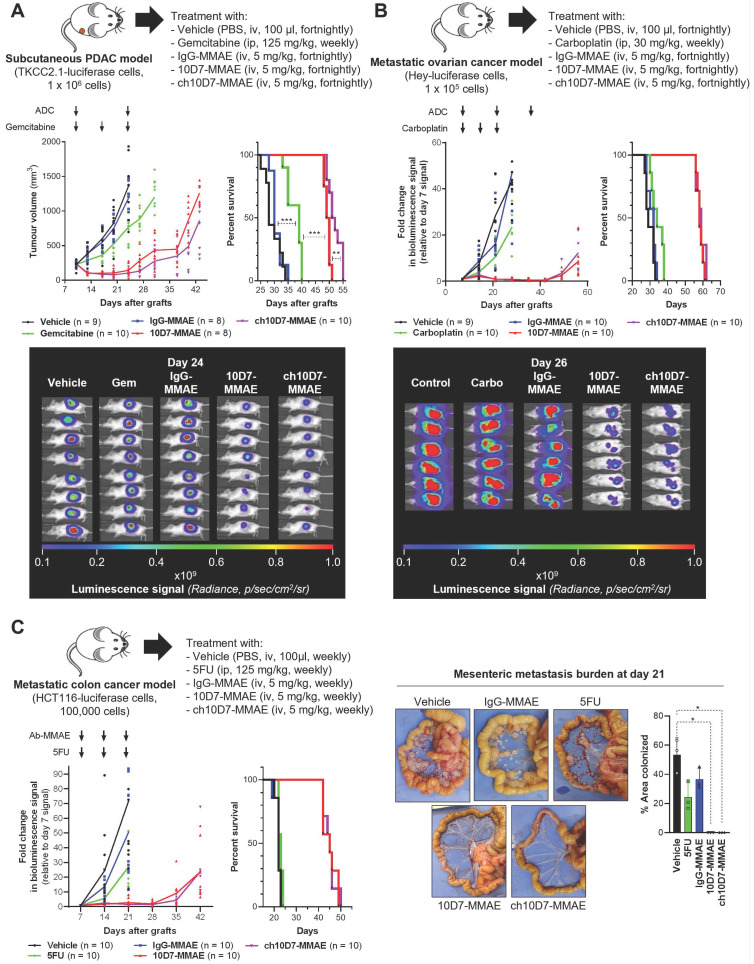
** Efficacy of ADC ch10D7-MMAE in *in vivo* cancer models.** Presented for each model: Top, Schematic of the experimental protocol including xenograft site, cancer cell line and treatment regimen; Bottom left: Graph of tumor burden versus time for each treatment group; Bottom right, Kaplan-Meier survival curve of mice in each treatment group. **A.** Preclinical model of PDAC involving subcutaneous xenografts in NSG mice of TKCC2.1 cells (1x10^6^/mouse; 8-10 mice/group). Once tumors reached 200 mm^3^ mice were randomized then treated with ADCs every two weeks (5mg/kg i.v.), weekly gemcitabine (125mg/kg i.p.) or vehicle control. **B.** Preclinical model of ovarian cancer involving intraperitoneal xenografts in NSG mice of luciferase labelled HEY cells (1x10^5^; 9-10 mice/group). One week after injection of cells mice were randomized then treated with ADCs every two weeks (5mg/kg i.v.), weekly carboplatin (30mg/kg i.p.) or vehicle control. **C.** Preclinical model of metastatic colorectal cancer involving intraperitoneal xenografts in NSG mice of luciferase labelled HCT116 cells (1x10^5^; 10 mice/group). One week after injection of cells mice were randomized then treated with ADCs every two weeks (5 mg/kg i.v.), weekly 5FU (125 mg/kg i.p.) or vehicle control. For subcutaneous xenografts, tumor burden was measured twice weekly using calipers, while tumor burden for intraperitoneal xenografts was measured by weekly bioluminescent imaging. Once mice in any group required euthanasia due to disease burden, treatments were stopped, and survival followed. Statistical significance of the survival analysis was assessed using Log-rank Gehran-Breslow Wilcoxon Chi^2^ test.

We next examined the efficacy of ch10D7-MMAE, in comparison with 10D7-MMAE and three standard-of-care chemotherapies, in *in vivo* models of pancreatic, ovarian, and colorectal cancer that express cell surface CDCP1 at levels above the identified threshold of 1×10^5^ receptors per cell (Figure 3D) and are responsive to ch10D7-MMAE *in vitro* (Figure 3B). As shown in Figure 5A, against subcutaneous xenografts of PDAC TKCC2.1 cells, two treatments with ch10D7-MMAE and 10D7-MMAE significantly slowed tumor growth in comparison with three treatments of the chemotherapy gemcitabine while growth of vehicle and IgG-MMAE treated tumors was uncontrolled. Kaplan-Meier analysis showed that median survival was 53 and 50 days, respectively, for ch10D7-MMAE and 10D7-MMAE treated mice, and only 39 days for gemcitabine treated mice and 32 and 30 days, respectively, for IgG-MMAE and vehicle treated mice (Figure 5A). Equally impressive results were seen in intraperitoneal xenograft mouse models of ovarian and colorectal cancer. Against ovarian cancer HEY cell xenografts, three treatments with the CDCP1 targeted ADCs almost completely blocked tumor growth for the duration of the assay, while three treatments of the chemotherapy carboplatin had little impact on tumor growth in comparison with vehicle and IgG-MMAE treated tumors the growths of which were uncontrolled (Figure 5B). Median survival was 60 and 59 days, respectively, for ch10D7-MMAE and 10D7-MMAE treated mice, and only 35 days for carboplatin treated mice and 32 and 31 days, respectively, for IgG-MMAE and vehicle treated mice (Figure 5B). Against colorectal cancer HCT116 cell xenografts, three treatments with ch10D7-MMAE and 10D7-MMAE significantly delayed tumor re-growth, while three treatments of the chemotherapy 5-fluorouracil (5FU) marginally slowed xenograft growth relative to control vehicle and IgG-MMAE treated mice (Figure 5C). The CDCP1 targeted ADCs impressively blocked colonization of the mesenteric membrane by HCT cells in comparison with 5FU and the control treatments (Figure 5C right). Median survival was 46 days for ch10D7-MMAE and 10D7-MMAE treated mice, and only 23 days for 5FU, IgG-MMAE and vehicle treated mice (Figure 5C). These results indicate that the ch10D7-MMAE ADC is effective *in vivo* against tumors that express cell surface CDCP1 above a threshold level of 1×10^5^/cell and display responsiveness *in vitro*.

### CDCP1 expression in cancer and normal human tissues

A key requirement for the efficacy of theranostic agents and ADCs is the elevated expression of the target on malignant above normal cells, such that a suitable signal-to-noise ratio is achievable for PET imaging and a therapeutic index can be attained for treatment 1-3, 19. To evaluate expression of CDCP1 in human cancer and normal human tissues, we examined its mRNA and protein levels in cohorts of normal and malignant samples. We first compiled CDCP1 mRNA expression profiles from datasets from the Pan-Cancer Analysis of Whole Genomes (PCAWG) Consortium, the Therapeutically Applicable Research to Generate Effective Treatments (TARGET) initiative and the Genotype Tissue Expression (GTEx) project 20-22. This allowed mRNA analysis of normal and tumor samples from 23 malignancies and 38 normal tissues, demonstrating that CDCP1 mRNA is overexpressed in all but seven of the cancers versus the corresponding normal tissues. Only adrenocortical carcinoma and cutaneous melanoma displayed mean transcript levels that were significantly lower in malignant versus normal tissues, while CDCP1 mRNA expression was not significantly modulated between normal and tumor in liver, prostate, rectum, thymus and uterus (Figure S5 Top). Highest CDCP1 mRNA expression in tumor versus corresponding normal was seen in cholangiocarcinoma versus bile duct, endometrioid carcinoma versus endometrium, ovarian serous cystadenocarcinoma versus ovary, and PDAC versus pancreas (Figure S5 top). Across all normal and malignant cohorts, highest levels of CDCP1 mRNA were observed in esophageal carcinoma, the normal head and neck region, head and neck squamous cell carcinoma, cervical and endo-cervical cancer, and PDAC (Figure S5 Bottom). Among the 20 highest expressers of CDCP1 mRNA, 16 were from cancer tissues and four from normal head and neck region (rank #2), rectum (rank #10), skin (rank #14) and thyroid gland (rank #20) (Figure S5 bottom). Also of note, high variability was seen in CDCP1 mRNA levels in normal bladder, breast, cervix, colon, esophagus, kidney and stomach (Figure S5) likely reflecting differences in CDCP1 levels between individuals.

**Figure 6:**
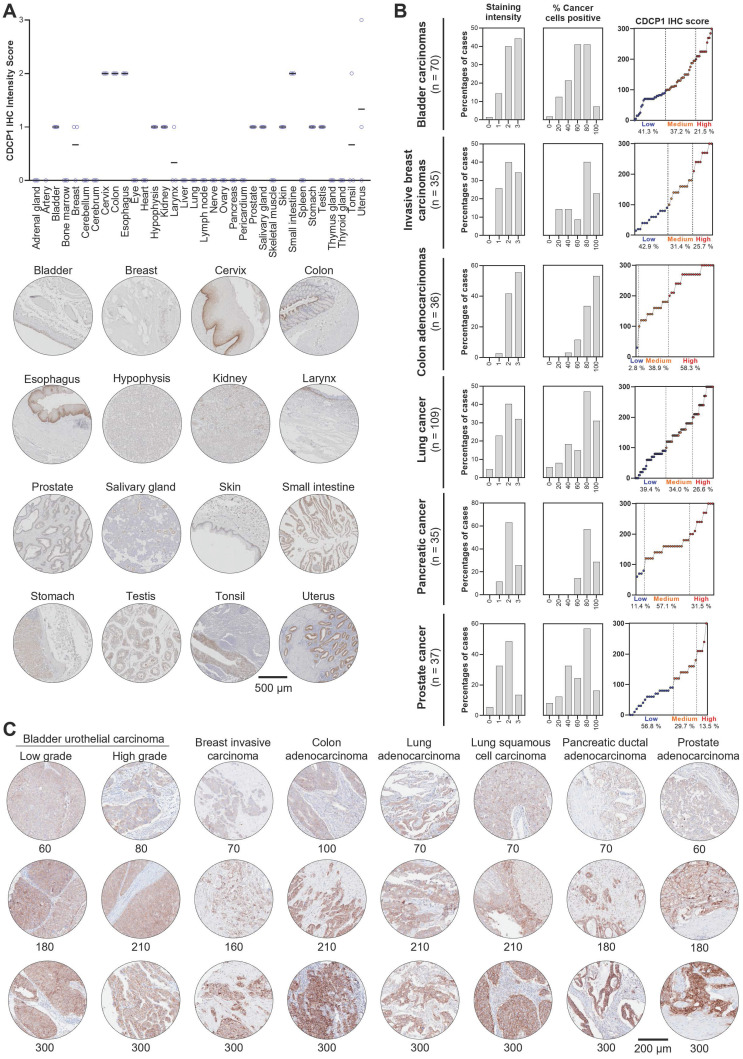
** CDCP1 expression in normal and malignant human tissues.** CDCP1 protein expression in normal human tissues and cancers determined by immunohistochemistry using antibody 4115. **A.** Top: Graph of immunohistochemistry staining intensity score (0 to 3). Black bar, mean staining intensity score; Circle, staining intensity score for each case. Bottom: Representative immunohistochemistry images of normal tissues for CDCP1 expression. For each tissue, the sample showing the highest staining intensity is shown. **B.** Graphs of scores for CDCP1 protein staining intensity (left) and percentage (%) cancer cell positive for CDCP1 protein (middle), and combined CDCP1 immunohistochemistry score (right) for six cancer types. **C.** Representative images of CDCP1 immunohistochemistry staining in various cancers for a range of immunohistochemistry scores.

To examine CDCP1 protein expression in human normal and cancer tissues, we performed Immunohistochemistry for CDCP1 using the commercial antibody #4115 which recognises a carboxy-terminal, intracellular epitope presents in the cell retained fragment and full-length CDCP1 12. If this antibody allows us to compare the level of CDCP1 expression in various tissues, it does not definitely demonstrate that CDCP1 will be targetable in those tissues using Ch10D7 which recognise an epitope located on the extracellular portion of CDCP1 protein 12. Nevertheless, the level of CDCP1 expression determined by IHC (IHC score) using the #4115 antibody on FFPE tissues from nine different cancer xenograft models showed a strong correlation with the number of antibodies bound per cell previously determined by quantitative flow cytometry using an antibody targeting the epitope recognised by ch10D7 (Figure S6). To examine normal CDCP1 protein expression, immunohistochemical analysis was performed on a tissue microarray containing 34 normal tissues with samples from 3 different individuals for each tissue. Of the 34 normal tissues, corresponding mRNA expression data were not available for only eye, larynx, pericardium and tonsil. Immunohistochemical staining was scored for “Staining Intensity” on a scale of 0 to 3 (0, no staining; 1, weak; 2, moderate; or 3 strong). As graphed in Figure 6A (top) and tabulated in Table 1, CDCP1 protein was undetectable in 18 of the 34 normal organs, and detected in 16 tissues, with representative images for each tissue in Figure 6A and S7 Eleven of the 16 CDCP1 expressing normal organs had low intensity staining (mean score ≤1; bladder, breast, hypophysis (pituitary), kidney, larynx, prostate, salivary gland, skin, stomach, testis, tonsil), five had highest intensity staining at intermediate levels (mean score >1 to 2; cervix, colon, esophagus, small intestine, uterus) while none displayed high mean intensity staining (>2). In expressing normal organs, CDCP1 protein was mainly detected on the plasma membrane of epithelial cells except in the hypophysis (pituitary) where cytoplasmic staining was observed in most expressing cells and could correspond to non-specific signal. Cytoplasmic CDCP1 staining was also detected in Sertoli cells and spermatocytes in the testis. In summary, immunohistochemical analysis showed that CDCP1 protein is restricted to a subset set of normal human tissues, with expression restricted to populations of mostly epithelial cells.

To assess whether CDCP1 protein is expressed at elevated levels in cancer tissues and examine inter- and intra-patient variability, we performed immunohistochemical staining of tissue microarrays containing 70 bladder carcinomas, 35 invasive breast carcinomas, 36 colon adenocarcinomas, 109 lung carcinomas, 35 PDACs, and 37 prostate adenocarcinomas. Staining was scored for “Staining Intensity” (0 - 3) and “Percentage of Cancer Cells Positive” (0 - 100), which were combined for an overall CDCP1 “Immunohistochemistry Score” and these three scores are graphed in Figure 6B for each of the six cancers, with representative images and associated immunohistochemistry scores shown in Figure 6C. As shown in Figure 6B, segregation of Immunohistochemistry Scores into “Low” (≤100), “Medium” (>100 to 200) and “High” (≥200 to 300) indicated that for five of the six malignancies more than 57% of tumors were medium to high CDCP1 expressers (58.7% of bladder cancers, 57.1% of breast cancers, 97.2% of colon cancers, 60.6% of lung cancers, 88.6% of PDACs) with the proportion of medium to high CDCP1 expressers in prostate cancer at 40.2%. Overall, the immunohistochemical data indicate that a significant proportion of bladder carcinomas, invasive breast carcinomas, colon adenocarcinomas, lung squamous cell carcinomas, PDACs and prostate adenocarcinomas display expression of CDCP1 protein that is markedly elevated above levels seen in normal tissues.

## Discussion

Novel treatments for advanced cancers are needed urgently. While five-year survival rates of low stage, resectable cancers are excellent, often exceeding 90%, outcomes for patients with metastatic disease continue to be poor owing to almost inevitable treatment resistance culminating in treatment failure for all but a small proportion of cases 23-25. While incremental improvements to conventional chemotherapy regimens and the addition of targeted small molecular weight drugs and biological agents are improving outcomes, the ability to achieve long-term remission for most metastatic cancers remains stubbornly elusive. The development of agents that target receptors enriched on cancer cells to deliver radio-imaging and cytotoxic payloads has the potential to improve on current outcomes by personalizing cancer management. Under this personalized approach, receptor-targeted radio-imaging via PET scan first selects patients whose tumors are enriched for the targeted receptor, and selected patients are then treated with receptor-targeted cytotoxic payloads 1-6.

The receptor CDCP1 is emerging as a target for theranostics and ADCs for multiple cancers including ovarian cancer, PDAC and castration resistant prostate cancer including PSMA null disease 12-15, 26, 27. Of note, elevated CDCP1 expression has been associated with the metastatic progression of pancreatic, breast and prostate cancers as well as of melanoma 26, 28-31. Here, we present the generation of chimeric anti-CDCP1 antibody ch10D7 and its characterization in terms of affinity and specificity for CDCP1 using biochemical and cellular assays. Consistent with the parent monoclonal mouse antibody 10D7 12, 14, 15, ch10D7 has high affinity and specificity for CDCP1 inducing its transient tyrosine phosphorylation and downstream signalling via Src followed by internalization of receptor/antibody complexes and CDCP1 degradation and release of antibody payloads. Consistent with these findings, ch10D7conjugated with the cytotoxin MMAE displays high anti-growth activity *in vitro* against cell lines derived from six different epithelial cancers. The potency of ch10D7-MMAE correlates with the level of cell surface CDCP1 above a threshold level of ~1×10^5^ receptors/cell (assuming each ch10D7 antibody has two receptor binding sites; based on data in Figure 3D). Cells with levels below this threshold are largely unresponsive to ch10D7, although several cell lines with expression above this threshold were also resistant to ch10D7-MMAE, including three clear cell renal cell carcinoma (ccRCC) cell lines, 786-O, A498 and ACHN, which display higher levels of cell surface CDCP1 than the threshold (Figure 3D). The mechanism of resistance in cells expressing cell surface CDCP1 above the threshold is unclear. Our western blot analyses indicate that ch10D7 causes degradation of CDCP1 in 786-O, A498 and ACHN cells (Figure 2D, S3), indicating that this antibody is able to bind to CDCP1 and be internalised to deliver a payload such as MMAE. ccRCCs commonly display resistance to systemically delivered chemotherapies and targeted therapeutics 32, and it is possible that ccRCCs are also resistant to MMAE released intracellularly by internalized ch10D7-MMAE.

Our findings from cancer xenograft models in mice and analysis of 34 normal tissues and cohorts of patient tumors, support CDCP1 as theranostic target to select cancer patients for CDCP1-directed therapies including ADCs. Our developed human/mouse chimeric antibody ch10D7 demonstrated efficacy at delivering payloads for detection and treatment of CDCP1 expressing cancer xenograft models in mice. ch10D7-^89^Zr was effective at detecting CDCP1-expressing PDAC xenografts in mice, and ch10D7-MMAE displayed robust anti-tumor effects *in vivo* against xenografts of PDAC, and ovarian and colon cancer, significantly prolonging mouse survival in comparison with controls including three chemotherapeutic agents widely used clinically.

While these findings from preclinical models indicate that CDCP1-targeted agents can be highly effective, our detailed expression analyses suggest that clinical implementation of CDCP1-targeted therapies, will benefit from the availability of an assay that is able to segregate patients that express CDCP1 at levels significantly elevated in tumors above expression in normal tissues. A significant proportion of the bladder (58.7%), breast (57.1%), colon (97.2%), lung (60.6%) and prostate (40.2%.) cancer and PDAC (88.6%) tumors analyzed in this study exhibited CDCP1 immunohistochemical signal well above levels seen by us in 34 normal tissues, suggesting that these patients would most likely benefit from a CDCP1-targeted therapy. However, our analyses also indicated that some individuals express CDCP1 at normal sites at levels that could be adversely impacted by a CDCP1-targeted therapy. Our mRNA expression analysis revealed high variability in CDCP1 mRNA levels in normal bladder, breast, cervix, colon, esophagus, kidney and stomach, and our immunohistochemical results indicated CDCP1 protein at intermediate levels on the epithelium of normal cervix, colon, esophagus, small intestine and uterus, suggesting these normal organs as sites for potential off-tumor effects. It is important to note that the antibody we used to assess CDCP1 protein expression in tissues (#4115) recognizes a different epitope to the ch10D7 antibody. Antibody 4115 recognizes a carboxyl-terminal epitope and thus demonstrates whether CDCP1 is oriented on the cell surface with its amino-terminal region located extracellularly to be available for binding of antibody ch10D7 which recognizes an epitope present within amino acid 30 to 358 of CDCP1 12, 15. However, the level of expression observed by immunohistochemistry cannot be used to directly predict ch10D7 binding capacity in the sample analyzed but only inform on CDCP1 protein expression levels and its localization on the plasma membrane. Together, our CDCP1 expression results suggest that clinical implementation of CDCP1 directed therapeutic agents will be facilitated by the availability of a screening protocol, such as molecular PET imaging, that is able to select cancer patients who have CDCP1 protein levels that are sufficiently elevated in tumors above normal organs.

The requirement to stratify cancer patients for receptor-targeted therapy is well recognized for PSMA which is widely expressed in normal human tissues, including bladder, kidney, testis, ovary, fallopian tube, breast, adrenal gland, liver, esophagus, stomach, small intestine, colon, and brain, as well as in hyperplastic prostate, Barrett's esophagus, and tumors of the prostate, bladder, kidney, testis, esophagus, stomach, small intestine, colon, adrenal gland, and lung 33. For PSMA, stratification is achieved by PSMA-targeted radio-imaging via PET scan, and our preclinical results indicate similarly that CDCP1-targeted PET imaging will be effective at selecting patients for CDCP1-targeted therapies. In addition, our flow cytometry results suggest that *ex vivo* evaluation of levels of cell surface CDCP1 in tumor biopsies or circulating malignant cells could also serve to stratify patients for CDCP1-targeted therapies. In addition, because we and others have shown that a region of CDCP1 can be shed from the cell surface and detected in colon cancer patient serum and function as a predictive biomarker of lung cancer onset 11, 34, 35, the detection of cell-shed CDCP1 may be suitable for selection of cancer patients suitable for CDCP1-targeted therapies.

In summary, our work reinforces the growing literature indicating that receptor CDCP1 is a rational target for the development of receptor-targeted agents for detection and personalized treatment of a range of cancers. The present work also provides a pipeline for assessing the suitability of other receptors as targets for cancer directed theranostics and ADCs.

## Material and methods

### Cell Lines and Culture Conditions

786-O, A498, 769P, A704, ACHN, Caki-1 and Caki-2 kidney cancer lines, SW620, SW480, HT29 and HCT116 colorectal cancer lines, CRL5889, H1299, A549, HCC827, H1975, H1650, EBC-1, HTB182 and H460 lung cancer lines, OVMZ6, A2780, SKOV3, PEO14, KURAMOCHI, OVSAHO, OV90, OVCAR3, OV93, PEO1, PEO4, CAOV3, OVCAR420, and HEY ovarian cancer lines, as well as PC3 and DU145 prostate cancer lines, were from the ATCC (Manassas, VA, USA) and cultured according to supplier protocols. The APGI PDAC patient derived cells TKCC10, TKCC09, TKCC27, TKCC15, TKCC23, TKCC05, and TKCC2.1 were cultured as described previously 12. Additional ovarian cancer cell lines TOV112D, TOV21G, OV93 and OVTOKO were kindly provided by Dr Katherine Roby (University of Kansas School of Medicine, Kansas City, KS), and KK and KOC7C cells by Dr. Hiroaki Itamochi (Tottori University School of Medicine, Yonago, Japan) and cultured as described previously 36. Using a previously described protocol 12, 15, 28, TKCC2.1, HEY and HCT116 cells were transduced with a luciferase expression construct. TKCC05 cells were stably transduced with lentiviral CDCP1 silencing constructs (shCDCP1 #1, shCDCP1 #2) or a scramble control construct (shControl) as described 28. A549 cells were stably transduced with a pLenti-PGK-Hygro-DEST expression vector (w530-1, Addgene) encoding CDCP1.

### SPR Analysis

Binding affinity of 10D7, ch10D7, 10D7-MMAE and ch10D7-MMAE to CDCP1-ECD was assessed as previously described using a Biacore T200 system (GE Healthcare) 36. Binding kinetics to antibodies immobilized on a Protein G sensor chip (Cytiva, Marlborough, MA) was of serial dilutions of the purified recombinant extracellular domain of CDCP1 (CDCP1-ECD; serial dilutions: 25 - 0.78 nM; 30 μL/min) with 120 s of association and 600 s of dissociation time at 25 °C. Data were processed using BIAevaluation software (GE Healthcare) with signals double-referenced by subtraction of a “buffer only” channel against the reference-subtracted sensorgrams. Kinetic data was obtained by globally fitting to a 1:1 binding model.

### Antibody radiolabelling with ^89^Zr and PET/CT imaging

ch10D7 and 10D7 were labelled with the positron-emitting radionuclide ^89^Zr as described 12, 14, 15. Yield and purity of the labelled antibodies were determined by radio-thin layer chromatography and high-performance liquid chromatography (Agilent, Mulgrave, Australia). PET-CT imaging was performed on NSG mice carrying subcutaneous PDAC cell xenografts (luciferase labelled TKCC2.1 cells). Two weeks after cell inoculations, mice received equivalent doses of the relevant ^89^Zr labelled antibody via the lateral tail vein (~4 MBq). PET-CT imaging was performed on isoflurane anaesthetised mice after 24, 48, 72 and 144 h using an Inveon PET/CT unit (Siemens, Munich, Germany). PET acquisition (30 minutes; static emission) was performed, and images were reconstructed using an ordered-subset expectation maximization (OSEM2D) algorithm, with CT attenuation correction. The CT scan parameters were 80 kV, 500 µA, 230 ms exposure time, 360° rotation with 180° rotation steps, binning factor of 4, low magnification position, producing an effective pixel size of 106 µm, with CT images reconstructed using the Feldkamp algorithm. All PET and CT images were reconstructed using Inveon Acquisition Workplace software (Siemens). PET activity per voxel was converted to Bq/cm^3^ using a conversion factor obtained by scanning a cylindrical phantom filled with a known activity of ^89^Zr to account for PET scanner efficiency. Activity concentrations within tissue regions of interest were expressed as percentage of the decay-corrected injected activity per cm^3^ of tissue (%ID/cc^3^; SUV) using Inveon Research Workplace software (Siemens). *Ex vivo* bio-distribution was assessed after the final imaging time point. Harvested tumor and organs, cleaned of blood, were weighed and radioactivity quantified using a Wizard 2480 gamma counter (Perkin Elmer) and presented as %ID/g of tumor or tissue (after decay and detector efficiency corrections).

### Antibody conjugation with MMAE

To conjugate ch10D7, 10D7 and IgG1κ with MMAE, antibody inter-chain disulfides were first partially reduced using DTT (10 nM, 15 min, 37°C) to generate free thiols, which were reacted with excess maleimide activated MC-VC-PAB-MMAE in 10% DSMO for 2 h at 37 °C. Reaction impurities were removed from crude reaction mixtures by filtering through Amicon Ultra Centrifugal Filters (Sigma-Aldrich). The drug-antibody ratio (DAR) of purified MMAE-labelled antibodies was determined by reverse phase LC/MS analysis of separated light and heavy chains as reported and determined to be 4.5 to 4.7 12, 15.

### Flow cytometry analysis

To compare the binding of ch10D7 and 10D7 to CDCP1-expressing cells, flow cytometry analyses were performed using IgGk1-550, 10D7-550 and ch10D7-550. TKCC05 and HEY cells were detached non-enzymatically using versene and blocked in PBS / 0.5 % BSA (30 minutes; 4 °C) before incubation of known numbers of cells with antibodies for 1h at 4 °C. After three washes using cold PBS, cells were then analysed by flow cytometry using a FACS Fortessa cytometer (BD Biosciences). To quantify the number of cell surface CDCP1 receptors, flow cytometry analyses were performed using anti-CDCP1 antibody CD318-PE and a standard curve generated using dilutions of a known concentration of PE-Quantibrite Beads (BD Biosciences, Hamilton, Australia). Cells detached non-enzymatically were blocked in PBS/0.5% BSA (30 minutes; 4 °C) before incubation of known numbers of cells with antibody CD318-PE (1 μM) which were then analysed by flow cytometry using an FACs Fortessa cytometer (BD Biosciences). The corresponding MFI value was used to interpolate the number of anti-CDCP1 antibodies per cell from a standard curve of the log_10_ values for the number of PE molecules per Quantibrite bead against the log_10_ of the corresponding MFI values.

### Real-time internalization assays

Internalization and degradation of CDCP1 on antibody binding, was examined using antibodies labelled with a Fab fluor pH sensitive dye. Diluted antibodies (10 µg/ml) were labelled by incubation in phenol red free complete medium containing the same molar concentration of Fabfluor-pH Antibody Labeling Dye (Sartorius, Dandenong, Australia) for 15 min at 37°C in the dark. TKCC05 (control shRNA or shCDCP1) and A549 (control vector or CDCP1 encoding construct) cells plated overnight in a black glass bottom 96 well plate (5,000 cells per well) were washed with PBS then incubated in phenol red free complete medium (50 µl) containing the relevant labelled antibody (5 µg/ml). Fluorescent signal per cell was acquired in real time using an Incucyte S3 system (Sartorius) imaging the plate every 15 min. Average fluoresence per cell was graphed against time.

### Statistical analysis

*In vitro* assays were performed in triplicate on three independent occasions. Analyses used GraphPad Prism (GraphPad, La Jolla, CA) with data displayed as mean and standard error of the mean (SD) except when indicated. Statistical significance was assessed by Two-way ANOVA or Student's t-test for parametric data, and for non-parametric data the Mann-Whitney test (t-test) or Kruskal-Wallis ANOVA, with *P-value ≤ 0.05, **P-value ≤ 0.01 and *** P-value ≤ 0.001.

## Supplementary Material

Supplementary materials and methods, figures.

## Figures and Tables

**Table 1:**
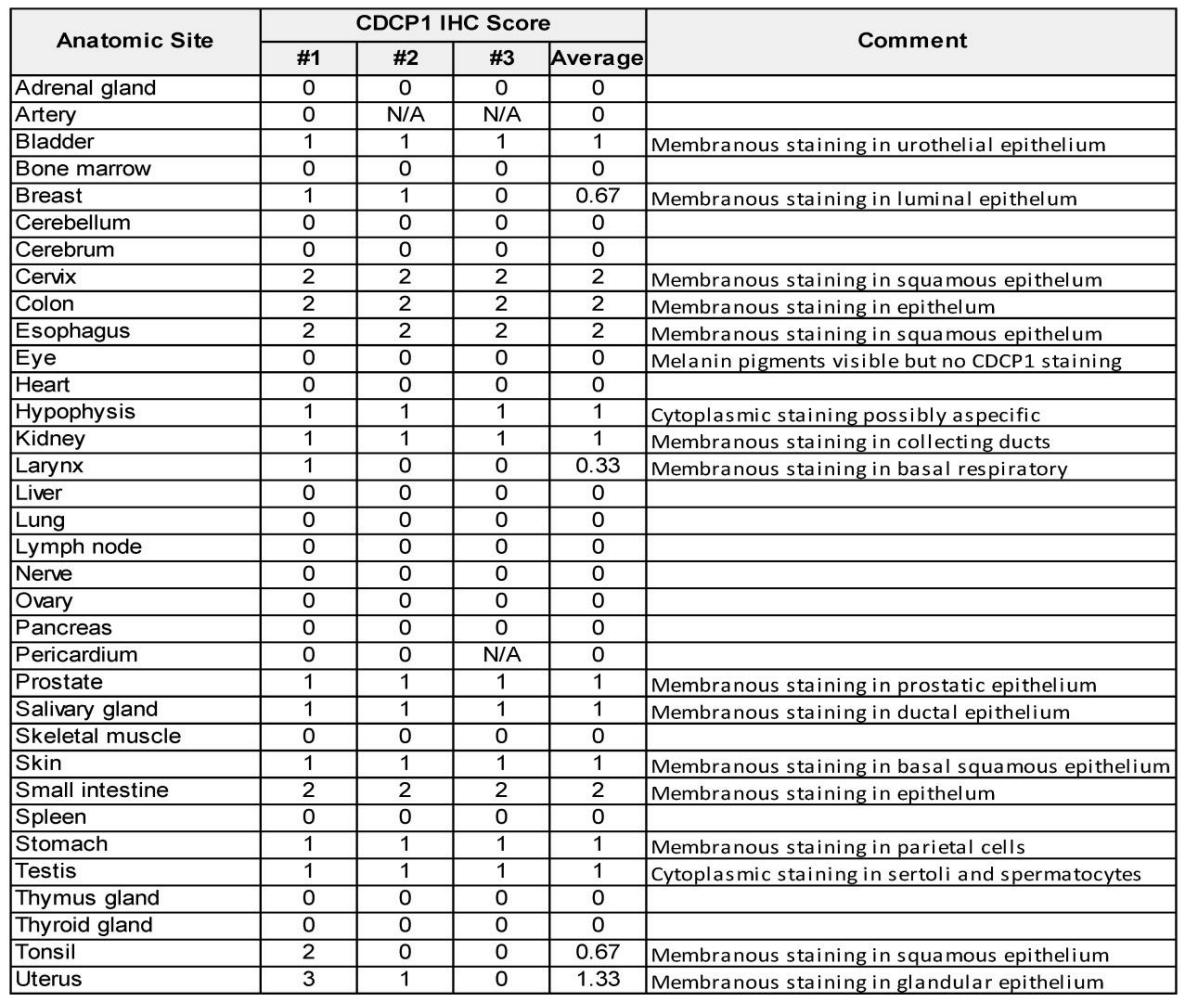
CDCP1 protein expression levels in normal human tissues

CDCP1 protein expression examined by immunohistochemical analysis of a tissue microarray containing 34 normal human tissues using anti-CDCP1 antibody 4115. Samples from three individuals were arrayed for each tissue (#1, #2, #3) and staining intensity scored (0 to 3) by an anatomical pathologist (CES).
