# Supplementary material for: CUB Domain-Containing Protein 1 (CDCP1) is a rational target for the development of imaging tracers and antibody-drug conjugates for cancer detection and therapy
Source: Theranostics. 2022 Oct 3;12(16):6915–30. doi: 10.7150/thno.78171 (PMC9576610; doi:10.7150/thno.78171)
Supplement: Supplementary file 1 — Supplementary materials and methods, figures. [file thnov12p6915s1.pdf]

SUPPLEMENTARY MATERIAL and METHODS

Reagents and Antibodies

10D7 was generated and purified from hybridoma conditioned media as previously described [37]. Anti-CDCP1 antibody #4115, anti-P-CDCP1-Y734 (#9050), anti-P-Src-Y416 (#2101) anti-Src (#2110), anti-GAPDH (#D4C6R), anti-rabbit IgG (H+L) DyLight™ 680 conjugate (#5366), and anti-mouse IgG (H+L) DyLight™ 800 4X PEG Conjugate (#5257) were from Cell Signaling Technologies (Genesearch, Arundel, Australia). Phycoerythrin (PE) tagged anti-CDCP1 antibody CD318-PE was from BioLegend (#324006; Karrinyup, Australia). PE Quantibrite beads (#340495) were from BD Biosciences (North Ryde, NSW). Atto-550 protein labeling Kit was from Sigma-Aldrich (St. Louis, MO). Recombinant CDCP1-ECD was generated and purified as previously described [11]. The maleimide activated drug linker, incorporating MMAE for generation of ADCs, was maleimidocaproyl-valine-citrulline-p aminobenzoyloxycarbonyl-MMAE (MC-VC-PAB-MMAE) and was purchased from Levena (San Diego, CA). Novolink Polymer detection kit (RE7150-CE) was from Leica Biosystems (Melbourne, Australia). Isotype control IgG1κ and EDTA immunohistochemistry buffer (E1161) were from Sigma-Aldrich (Castle Hill, Australia). Wheat Germ Agglutinin, Alexa Fluor™ 488 Conjugate (WGA-488) and DAPI were from Thermofisher (Seventeen Mile Rocks, Australia)

Expression and purification of antibodies

The ExpiCHO-S expression system (ThermoFisher Scientific, Waltham, MA USA) was used for transient ch10D7 expression. CHO cells were cultured in ExpiCHO-S Expression Medium (Gibco™, ThermoFisher Scientific) and transient transfection with an equimolar ratio of each of the vectors encoding the heavy chain and light chain of

ch10D7. After seven days of culture, the conditioned media was centrifuged at 4000*g* for 10 min at 4°C then the supernatant was filtered (0.22 µm pore size). Antibody was purified from the clarified media by immunoaffinity chromatography on an ÄKTA pure protein purification system (Cytiva, Marlborough, MA, USA) using 5 a ml HiTrap MabSelect SuRe column (Cytiva), then desalted on a HiPrep 26/10 desalting column (Cytiva). After separation of the protein isolate by electrophoresis, ch10D7 purity was quantified by densitometric analysis of the Coomassie stained SDS-PAGE gel and its concentration was determined using a Nanodrop spectrophotometer (Thermofisher Scientific).

Antibody labelling with Atto-550

Labelling of human IgG1k, 10D7 and ch10D7 with fluorophore Atto-550 was performed following the instructions of the manufacturer. Briefly, antibodies were diluted to 2 mg/mL in PBS and incubated with the reactive Atto-550 dye (solubilized in DMSO) for 2 h at room temperature in the dark with gentle agitation. Labelled antibodies were separated from free excess dye using a gel filtration column with a size exclusion limit of 5 kDa. Columns were equilibrated with PBS before sample loading then elution of the labelled antibody with PBS. Eluted antibody concentrations were determined by BCA assay.

Immunofluorescent analysis

TKCC05 cells plated in 96 wells plate (5,000 cells per well) were washed with PBS then incubated in complete medium containing 5 µg/ml of IgG-550, 10D7-550 or ch10D7-550. Cells were fixed at defined time points (5 – 120 min) using 4% PFA solution (15 min at room temperature), PBS washed and incubated with a solution of

PBS containing DAPI (1:10,000) and WGA-488 (1:1,000) for 30 min at 4°C. After three washes, cells were imaged using an inverted fluorescent microscope.

Lysate preparation, immunoprecipitation and Western blot analysis

Cells were lysed in RIPA buffer containing EDTA-free Complete protease inhibitor (1x), sodium vanadate (2 mM) and sodium fluoride (10 mM). Lysates were homogenized by passing the samples through 26-G needles and cleared by centrifugation at 14,000 g and 4°C for 30 min. Protein concentration was quantified by micro-bicinchoninic acid assay (Thermo Fisher Scientific). Lysates were separated by SDS-PAGE under reducing conditions (except where noted), transferred onto nitrocellulose membranes, and blocked using fish gelatin blocking buffer (3% w/v in PBS). Membranes were incubated with primary antibodies diluted in blocking buffer overnight at 4°C, washed with PBS containing 0.1% Tween 20, and then incubated with appropriate secondary antibody. Signals were detected using an Odyssey Imaging System and software (LI-COR Biosciences, Millennium Science, Mulgrave, Australia).

Mining of mRNA expression datasets

RNA sequence and pathology data from the PCAWG Consortium dataset of 2,658 cancers across 38 tumor types [21], the TARGET initiative of 9 types of childhood cancer from 6,319 participants [22] and the GTEx dataset (version 8) of 15,253 RNA-sequenced samples of 54 non-diseased tissue sites from 838 donors [20] was analyzed using the online Xena Functional Genomics Explorer [38]. The RNA-seq data in $\log_2$ Fragments Per Kilobase of transcript per Million mapped reads (fpkm) were

exported and used to generate box plots of median mRNA expression ± 10-90 percentile.

Immunohistochemistry

Immunohistochemistry was performed with anti CDCP1 antibody 4115 (1/200 dilution; overnight at 4°C) using a Novolink Polymer detection kit (Leica Biosystems, Mount Waverley, Australia) as described previously [12]. Commercial Tissue Microarrays (US Biomax) were used including: Multiple organ normal tissue microarray, 34 types of normal human organs, 99 cores, each type taken from 3 different individuals (FDA999x); Multiple organ carcinoma tissue microarray, containing 38 each cases of breast invasive carcinoma, lung squamous cell carcinoma, colon adenocarcinoma, prostate adenocarcinoma and pancreas adenocarcinoma, single core per case (BC000119b) ; Multiple bladder carcinoma with bladder tissue microarray, containing 53 cases of urothelial carcinoma, 11 adenocarcinoma, 5 squamous cell carcinoma, 1 small cell neuroendocrine carcinoma (BL1501a) and Lung adenocarcinoma tissue (75 cases), clinical stage I, II, III, IV (HLugA150CS02). Staining was assessed by an anatomical pathologist (CES) blinded to clinical data and scored semi quantitatively as described previously [12, 15]. For normal tissues, each core was assigned a score for staining intensity only (0, no staining; 1, weak; 2, moderate; or 3 strong). For cancer tissues, each core was assigned a score based on staining intensity (0, no staining; 1, weak; 2, moderate; or 3, strong) multiplied by the percentage of positive cells (in 20% increments) to produce a combined score that was assigned an integer value of 0 (combined score 0), 1 (combined score 1 to 100), 2 (combined score 101 to 200) or 3 (combined score 201 to 300).

In vitro ADC cytotoxicity assays

To assess the cytotoxic activity of IgG-MMAE, 10D7-MMAE and ch10D7-MMAE, cells (5,000/well) were plated in 96-well plates and allowed to attach for 24h [12, 15]. Cells were then treated for 6 h with respective ADC at indicated concentration before to be washed with complete medium and incubation for another 72 h. Relative cell viability was then measured by adding CellTiter AQueous One Solution Reagent to each well and measuring absorbance at 490 nm.

For colony forming assays, cells were plated in 6 wells plate at a density of 100,000 cells per well and allowed to attach for 24h. Cells were then treated for 6 h with respective ADC at indicated concentration before to be washed with complete medium, detached using a non-enzymatic solution and centrifuge at 370g for 5 min. Cell pellets were resuspended at a density of 500 cells/ml in complete medium and seeded in 24 wells plate. After 10 – 14 days, cells were fixed using 4% PFA solution and stained with 4% crystal violet solution in methanol (w/v).

In vivo testing of ADCs

In vivo assessment of ADC efficacy was performed on luciferase labelled TKCC2.1 PDAC cells, HEY ovarian cancer cells, and HCT116 colon cancer cells, with bioluminescent tumor burden measured weekly, like what was outlined previously [12, 15]. For the TKCC2.1 PDAC model, cells (1,000,000/mouse) were injected sub-cutaneously into the left flank of female NSG mice. Tumor volume was measured twice weekly (caliper measurement) and treatment started once tumors reached 200 mm^3, at which point mice were randomised into groups (8-10 mice per group) with equivalent tumor volumes. Mice were treated with either vehicle (PBS, iv, 100µl, fortnightly),

gemcitabine chemotherapy (i.p., 125 mg/kg weekly), or ADC (IgG-MMAE, 10D7-MMAE or hu/mu 10D7-MMAE, iv, 5mg/kg, fortnightly). Once mice in any group required euthanasia due to disease burden, treatment have been stopped and survival of mice have been followed.

For HEY ovarian cancer and HCT116 colon cancer experiments, female and male NSG mice respectively were injected intra-peritoneally with luciferase labelled HEY or HCT116 cells (100,000 cells per mouse). Tumors were allowed to grow for one week, after which time mice were randomised to groups (10 mice per group) with equivalent tumor burdens, and treatment begun with vehicle (PBS, iv, 100µl, fortnightly), chemotherapy (125 mg/kg weekly carboplatin for HEY cells I.P injection, 100mg/kg weekly 5FU for HCT116 cells I.P injection), or ADC (IgG-MMAE, 10D7-MMAE or hu/mu 10D7-MMAE, iv, 5mg/kg, fortnightly). Once mice in any group required euthanasia due to disease burden, treatment have been stopped and survival of mice have been followed. Additionally, for HCT116 mice, when the first mouse was culled (Day 24), we euthanized 3 mice per group to assess metastatic burden in the mesenteric membrane, a common site of intraperitoneal colorectal cancer metastasis, using a previously established protocol [39]. Mesenteric tumor burden was quantified from examination of the right colonic mesentery and quantified using ImageJ software (Fiji) to draw regions of interest and quantify the total surface area of the mesentery and the area replaced by tumor nodules.

SUPPLEMENTARY FIGURES

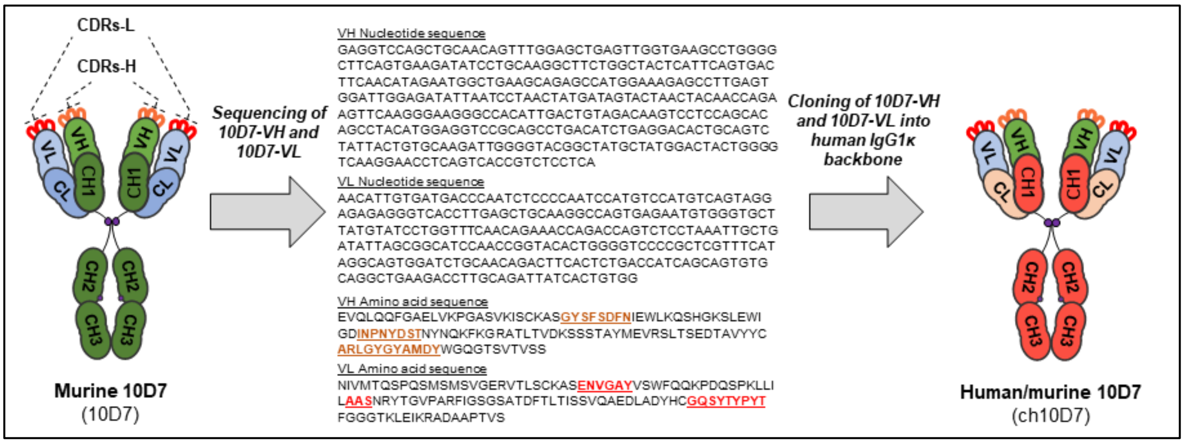

Supplementary Figure 1. Sequences used to develop ch10D7. Schematic illustrating the generation of human/mouse chimeric anti-CDCP1 antibody ch10D7 from murine 10D7, and nucleotide and amino acid sequences of VL and VH chains of the chimera are presented with CDRs highlighted in orange on the VH amino acid sequence and in red in the VL amino acid sequence.

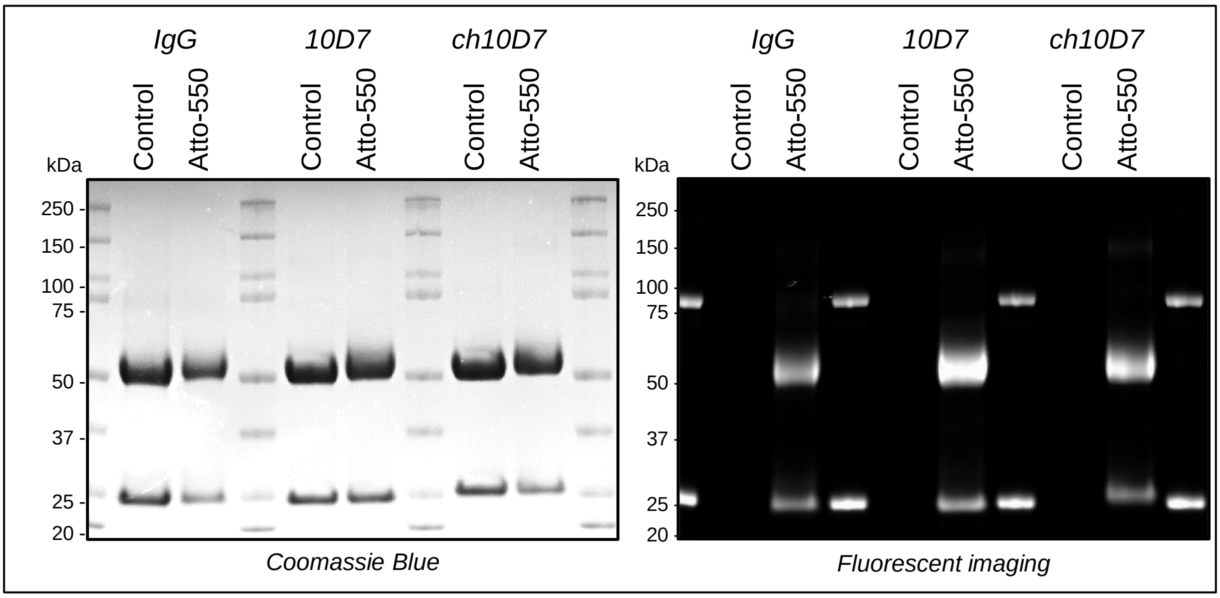

Supplementary Figure 2. SDS-PAGE analysis of fluorescently labelled antibodies. Control IgG, 10D7 and ch10D7 antibodies were analysed by SDS-PAGE followed by Coomassie blue staining and fluorescent imaging before and after conjugation with the Atto-550 fluorescent dye.

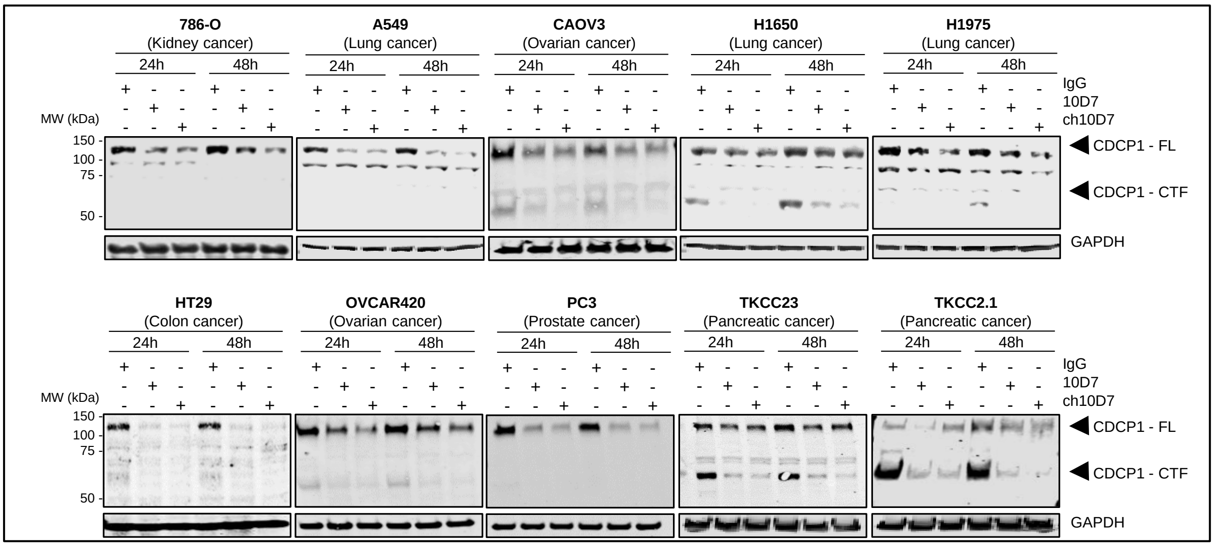

Supplementary Figure 3. Antibody-induced CDCP1 degradation in cancer cell lines. Impact of anti-CDCP1 antibody on CDCP1 receptor expression in cancer cells. Western blot analysis of lysates from 10 different cancer cell lines treated for 24 h or 48 h with 10D7, ch10D7 or control IgG (5 µg/ml). Lysates were probed for CDCP1 (antibody 4115) and GAPDH.

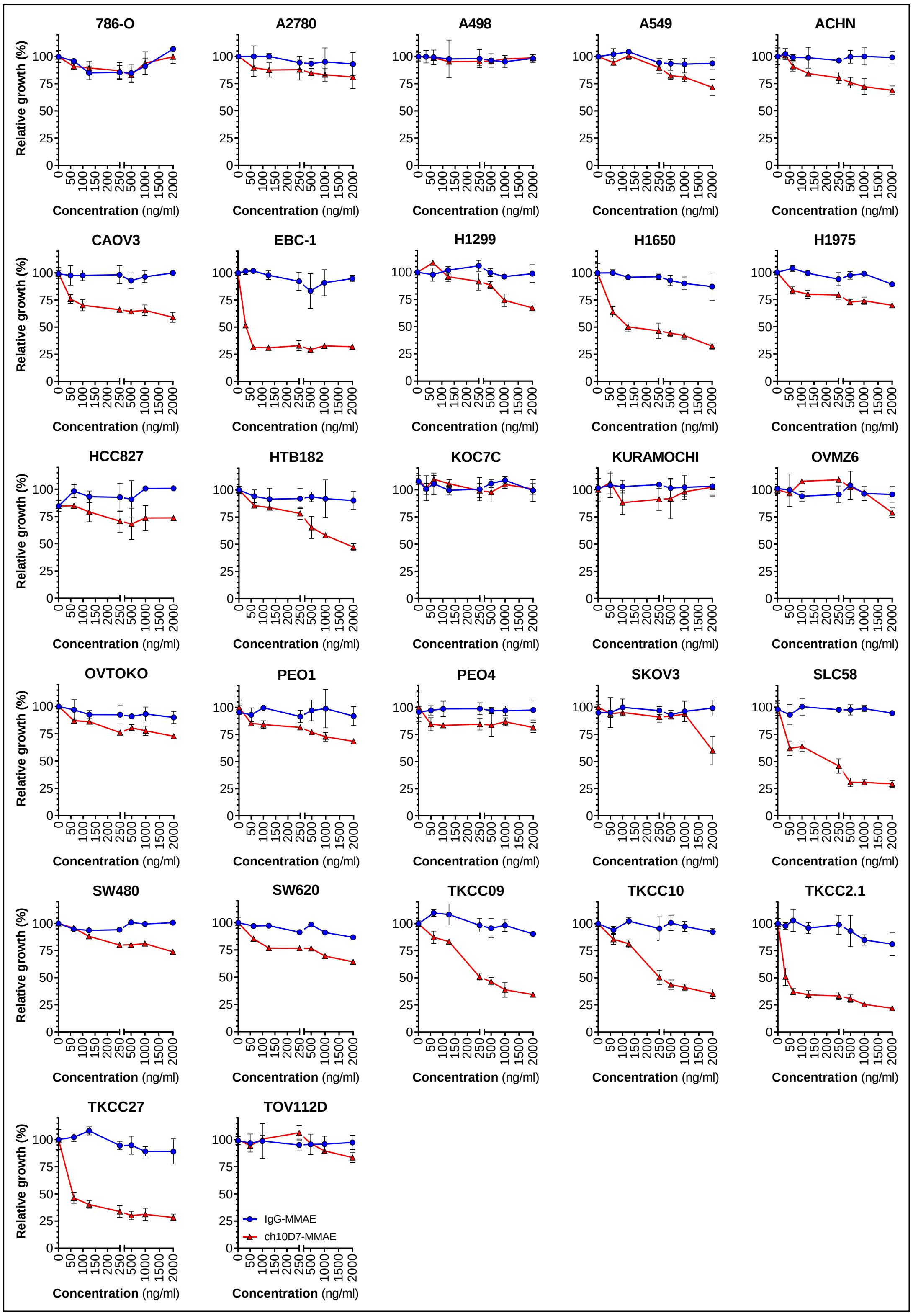

Supplementary Figure 4. Growth inhibition of cancer cells by ADCs. Cancer cell lines (4,000 cells/well) were treated for 6 h with the respective ADC (0 – 2000 ng/ml) then grown for a further 72 h in complete medium. Cell growth was quantified by absorbance measurements at 490 nm of wells incubated with the CellTiter AQueous One Solution Reagent. Data are presented as mean of relative cell growth (compared to untreated cells) +/- SD from 3 independent experiments.

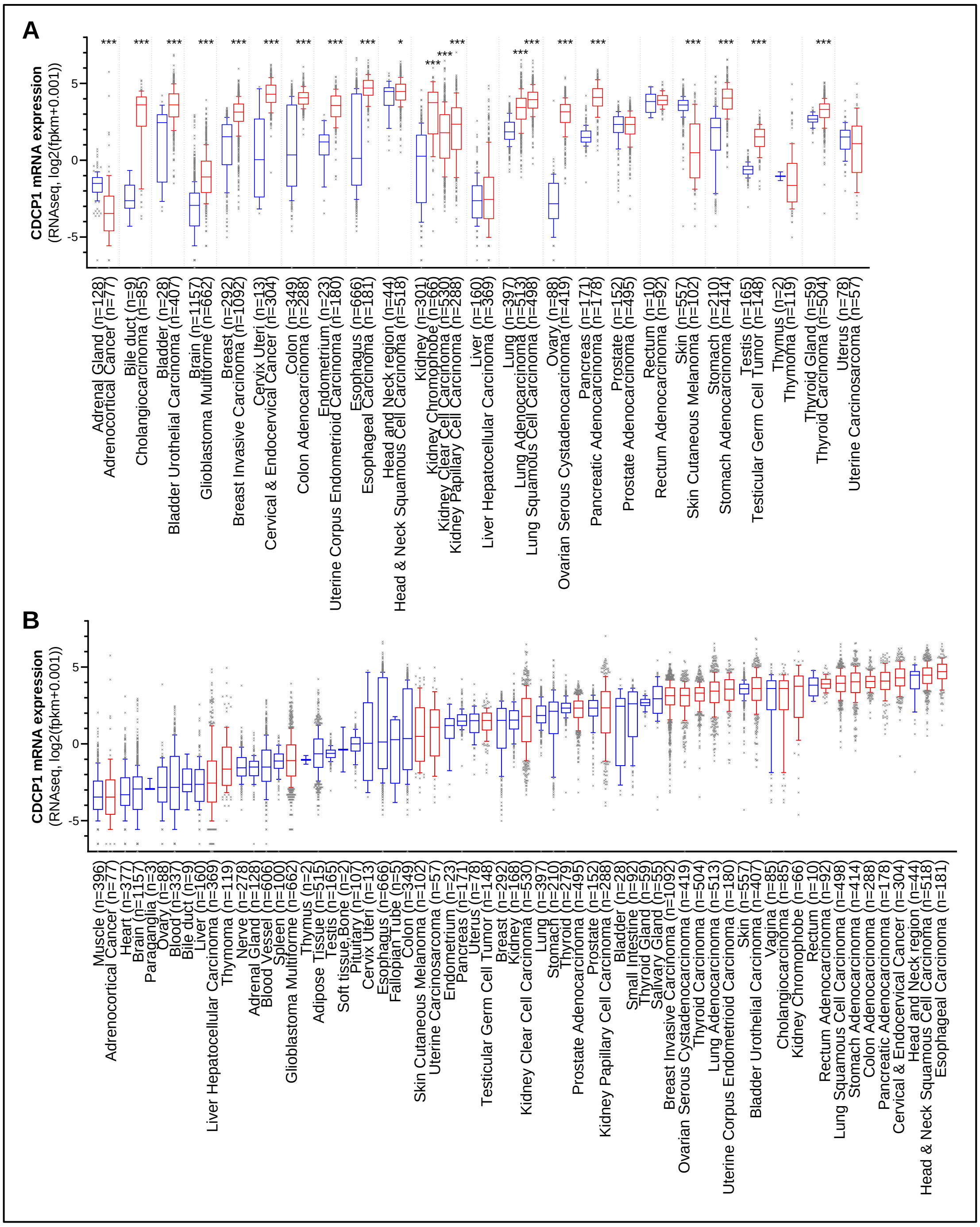

Supplementary Figure 5. CDCP1 mRNA expression in normal and malignant human tissues. A. Graph of CDCP1 mRNA in normal/tumor pairs for 23 cancer types. **B.** Graph of CDCP1 mRNA in normal/tumor pairs for 23 cancer types and 15 other unpaired normal tissues with sites arranged left to right from lowest to highest CDCP1 mRNA level. Level of expression is represented as median +/- 10-90 percentile Log_2(FKPM+0.001) of data extracted from PCAWG, TARGET and GTEx datasets using the online Xena Functional Genomics Explorer. Red, malignant tissues; Blue, non-malignant tissues; n, number of samples.

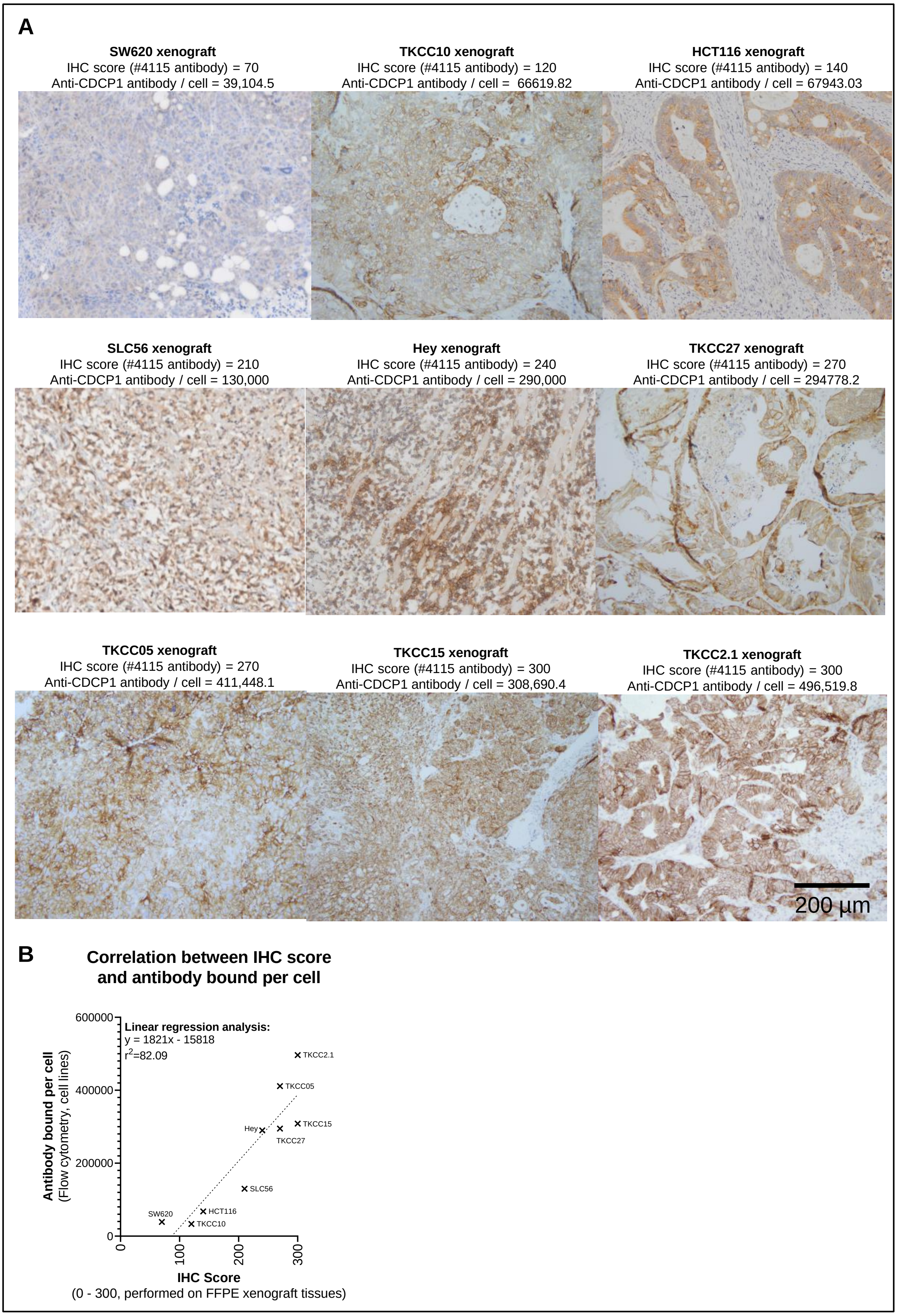

Supplementary Figure 6: Immunohistochemistry analysis of CDCP1 expression in tissues correlates with number of antibodies bound per cell. A. Representative image of CDCP1 Immunohistochemistry staining performed with #4115 antibody on 9

different xenograft tumours. Are indicated: Xenograft model; IHC score; Antibody bound per cell determined by flow cytometry using a commercial antibody recognizing the same epitope than ch10D7. **B.** Correlation between IHC score and number of antibodies bound per cell for each cellular model.

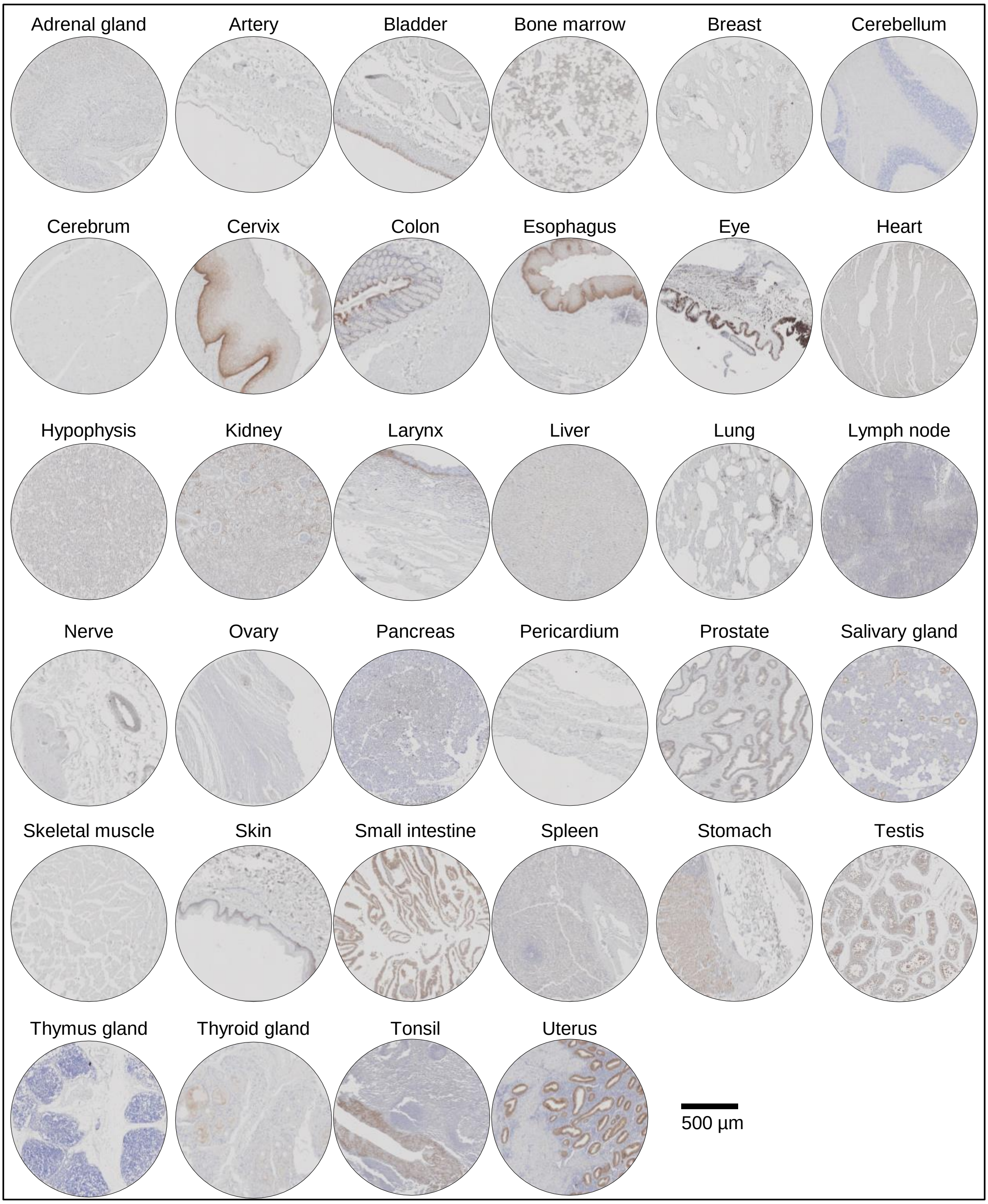

Supplementary Figure 7. Representative images of immunohistochemical staining for CDCP1 in normal human tissues. CDCP1 staining was performed using antibody 4115 on a tissue microarray containing cores from 34 normal human tissues (samples from 3 individuals per tissue). For each tissue type, the sample with the highest staining score is shown.
